# Supplementary material for: The Photoperiod-Driven Cyclical Secretion of Pineal Melatonin Regulates Seasonal Reproduction in Geese (Anser cygnoides)
Source: Int J Mol Sci. 2023 Jul 26;24(15):11998. doi: 10.3390/ijms241511998 (PMC10419153; doi:10.3390/ijms241511998)
Supplement: Supplementary file 1 [file ijms-24-11998-s001.zip › ijms-2469643-supplementary.pdf]

Table S1. Composition and nutrient content of experimental diet (air-dry basis).

Item	Content	Item	Content
Metabolic energy (MJ/kg)	11.29	Zinc (mg/kg)	33
Crude protein (%)	16	Calcium (g/kg)	45
Corn powder (g/kg)	403	Manganese (mg/kg)	55
Crushed wheat (g/kg)	250	Available phosphorus (g/kg)	35
Bean cake powder (g/kg)	135	Vitamin A (IU/kg)	4409
Green hay powder (g/kg)	127	Vitamin D3 (IU/kg)	661
Salt (g/kg)	5		

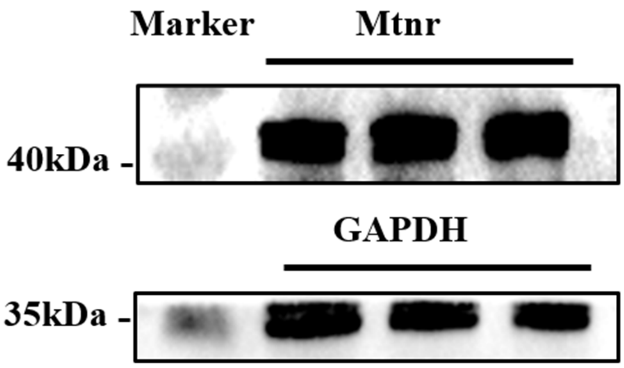

Figure S1. Verification of antibody specificity.
